# Correction to: Performance of a consensus-based algorithm for diagnosing anastomotic leak after minimally invasive esophagectomy for esophageal cancer

**DOI:** 10.1093/dote/doae056

**Published:** 2024-07-05

This is a correction to: Jobbe Lemmens, Bastiaan Klarenbeek, Moniek Verstegen, Frans van Workum, Gerjon Hannink, Sander Ubels, Camiel Rosman, Performance of a consensus-based algorithm for diagnosing anastomotic leak after minimally invasive esophagectomy for esophageal cancer, Diseases of the Esophagus, Volume 36, Issue 10, October 2023, doad016, https://doi.org/10.1093/dote/doad016

In the originally published paper, “Chemoradiation” and “Chemotherapy” were inadvertently switched in Table 1. This error has been corrected in the original paper.

